# Supplementary figures and images for: Optimal CD8+ T‐cell memory formation following subcutaneous cytomegalovirus infection requires virus replication but not early dendritic cell responses
Source: Immunology. 2021 Jun 13;164(2):279–91. doi: 10.1111/imm.13368 (PMC8442243; doi:10.1111/imm.13368)

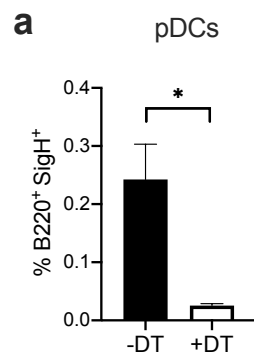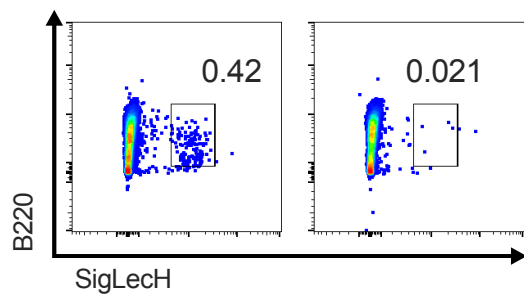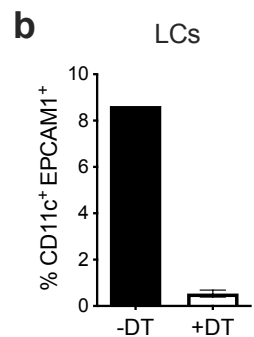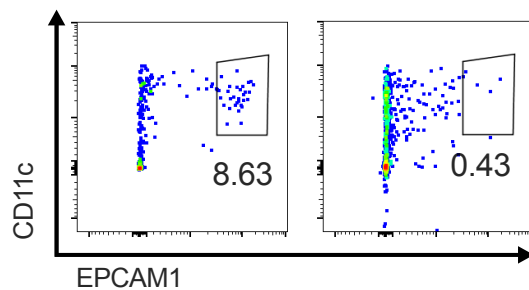

**c** cross-presenting DCs

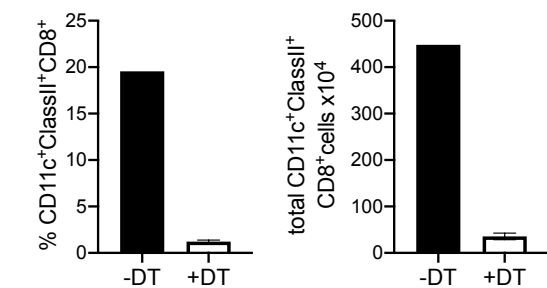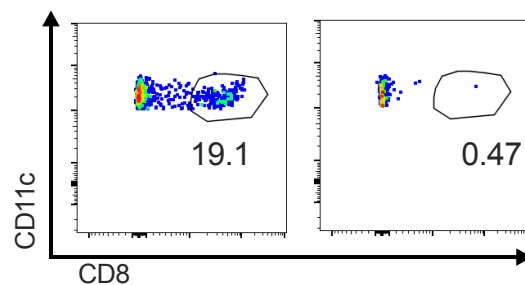

**d** classical DCs

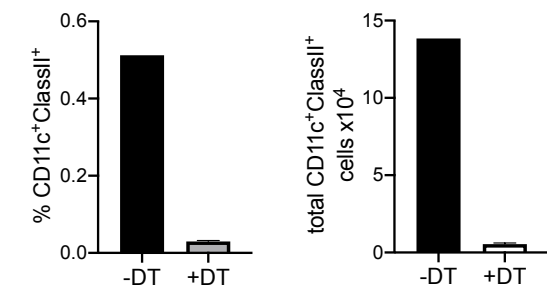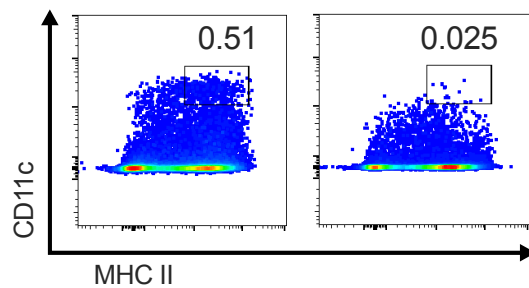

Supplement: Supplementary file 1 — Figure S1 [file IMM-164-279-s001.pdf]
